# The Cellular Proteins Grb2 and DDX3 Are Increased upon Human Cytomegalovirus Infection and Act in a Proviral Fashion

**DOI:** 10.1371/journal.pone.0131614

**Published:** 2015-06-29

**Authors:** Yolaine Cavignac, Diana Lieber, Kerstin Laib Sampaio, Johannes Madlung, Tobias Lamkemeyer, Gerhard Jahn, Alfred Nordheim, Christian Sinzger

**Affiliations:** 1 Institute of Medical Virology and Epidemiology of Virus Diseases, University of Tübingen, Tübingen, Germany; 2 Institute of Virology, University of Ulm, Ulm, Germany; 3 Proteome Center, University of Tübingen, Tübingen, Germany; University of San Francisco, UNITED STATES

## Abstract

While it is well established that human cytomegalovirus (HCMV) upregulates many cellular proteins and incorporates several of them into its virion, little is known about the functional relevance of such virus-host interactions. Two cellular proteins, Grb2 and DDX3, gained our interest as they appeared enriched in virion particles and this incorporation depended on the viral tegument protein pp65, suggesting a functional relevance. We therefore tested whether the level of these proteins is altered upon HCMV infection and whether they support viral replication. Immunoblotting analyses of cellular fractions showed increased levels of both proteins in infected cells with a maximum at 2 d p.i. and a reduction of the soluble Grb2 fraction. Knockdown of either gene by transfection of siRNAs reduced viral spread not only of the cell culture adapted HCMV strain TB40/E but also of recent clinical isolates. Apparently, Grb2 and DDX3 are proviral cellular factors that are upregulated in infected cells.

## Introduction

Infectious particles of the human cytomegalovirus (HCMV) are mainly composed of viral structural proteins including capsid, tegument and envelope proteins [1]. In addition, cellular proteins are incorporated to variable degrees [2–5]. Cellular virion components may simply reflect random inclusion at the site of maturation but may also act in a proviral or antiviral fashion [6–8]. Unlike alpha-herpesviruses, HCMV does not shut off the host protein synthesis but rather stimulates a plethora of cellular genes, some of which might be exploited for the sake of viral replication [9, 10]. On the other hand, cellular factors may be induced as part of the intrinsic defense [10, 11]. In either case, upregulation of a cellular protein upon infection is assumed to hint at a functional contribution during viral replication and pathogenesis [10–12].

Proteomic approaches have been successfully applied to analyze the composition of HCMV virions regarding viral and cellular proteins. While more than 70 cellular proteins were found associated with HCMV virions [3, 4], little is known about their role during viral replication. Here we report on two cellular proteins, Grb2 and DDX3, that were incorporated into virions depending on the viral tegument protein pp65, which suggested a functional relevance. Grb2 is a cytoplasmic adaptor protein that associates with activated epidermal growth factor receptor and thus contributes both to receptor-mediated signaling and to endolysosomal sequestration of the receptor [13, 14]. DDX3 is a multifunctional protein that shuttles between the cytoplasm and the nucleus, contributes to transcription, splicing, mRNA export and translation and is manipulated by a number of viruses including hepatitis C virus (HCV), hepatitis B virus (HBV), human immunodeficiency virus (HIV) and poxviruses [15].

These proteins were further analyzed regarding their expression levels during the replicative cycle of HCMV and their functional contribution to virus growth.

## Materials and Methods

Cells and Viruses: Human foreskin fibroblasts (HFFs) were isolated following written informed consent of the parents from tissue samples that were residuals from routine procedures (approved by the Ethics Committee of the Medical Faculty of Tübingen University; Ref. no: 339/2004A). HFFs were cultured in MEM (GIBCO/ Invitrogen) containing 5% FCS, 2.4 mmol/l glutamine, 100 μg/ml gentamicin and 0.5 ng/ml bFGF (basic fibroblast growth factor, GIBCO/ Invitrogen). For the knockout of UL83 in the genetic backbone of HCMV strain TB40-BAC4, a linear PCR fragment containing a kanamycin-resistance gene flanked by FRT sites and HCMV homology sequences was generated from plasmid pCP15 using primers 5’-TGCGGCGGGTGGCTCAACCTCGGTGCTTTTTGGGCGTCGAGGCGATGCATGCGGGGGTGTCCAGGGTTTTCCC-3’ and 5’-CGCGCAGGCAGCATGGAGTCGCGCGGTCGCCGTTGTCCCGAAATGATATCCTTCCGGCTCGTATGTTGTGTGG-3’. This fragment was inserted into TB40-BAC4 by homologous recombination in *E*. *coli*, thereby deleting the UL83 open reading frame except 34 bp downstream of the initiating ATG and 35 bp upstream of the terminating TGA as described previously. The resulting BAC was transfected into HFF for reconstitution of virus HCMV-TB40-BAC4-delUL83. Sequence data of the recombination sites (not shown) proved the correctness of the mutant. HCMV strains TB40/E [16], TB40-BAC4 [17], TB40-BAC4-delUL83 (see S1 Fig) and UL32-EGFP-HCMV-TB40 [18] were propagated in HFFs. For preparation of virus stocks, infectious supernatants from HFF cultures were harvested at days 5–7 post infection. Cellular debris was removed by centrifugation at 3220 x g for 10 min and the supernatants were either directly applied or stored at -80°C until used in experiments. The infectious titer in HCMV preparations was determined by limiting dilution assays [19] in fibroblasts on 96-well-plates using immunofluorescence staining of viral immediate early antigens as a readout.

Gradient purification of HCMV virions: For preparation of purified virions, HFF were infected at a multiplicity of infection (MOI) of 0.1. Supernatant of infected cultures was harvested at 6 days postinfection (p.i.) and cleared of cellular debris by centrifugation for 10 min at 2800 g. The viral particles were pelleted for 70 min at 80,000 g and resuspended in 1 ml 0.04 mol/l Na-phosphate buffer pH 7.4. For separation of the virions from dense bodies and NIEPS, this suspension was applied on a glycerol-tartrate gradient [20] and centrifuged for 45 min at 80,000 g and 10°C without deceleration. The purification was repeated two times. The purity of the virion preparations was controlled by electron microscopy after negative staining with uranyl acetate.

Western Blot: For comparison of Grb2 and DDX3 levels in virions and infected cells, protein samples from gradient purified virions and the respective producer HFFs were prepared according to standard protocols and the protein concentration was determined using the DC Protein Assay Kit (Bio-Rad). The samples were separated in 10% SDS PAGE gels and blotted onto nitrocellulose. Membranes were subsequently incubated with the respective primary and secondary antibodies. Primary antibodies were rabbit anti-HCMV-pUL26 antibody (kindly provided by T. Stamminger, University of Erlangen, Germany) [21], mouse anti-GRB2 antibody (BD Biosciences), rabbit anti-DDX3 antibody (Anaspec) and mouse anti-DDX3 antibody (clone AO196, kindly provided by A. Patel) [22]. Peroxidase-conjugated polyclonal rabbit anti-mouse Ig or swine anti-rabbit Ig sera (Dako) were used as secondary antibodies. Proteins were visualized with the Super Signal West Pico chemiluminescence substrate (Pierce), and bands were densitometrically analyzed using a Fluor-S MAX Multiimager with Quantity-One Software (Bio-Rad). The global amount of blotted proteins was measured by densitometric analyses of the Ponceau S staining, and each antigen-specific signal was then normalized to amounts of proteins in the respective lane.

For analyses of protein levels in subcellular fractions, cells were trypsinized, washed in ice-cold PBS and lysed with detergent buffer (PBS with 1% NP40 and 25 units/ml Benzonase) on ice for 10 min. NP40 is a mild detergent that does not disrupt nuclear membranes and does not destroy the cytoskeleton. A respective lysate will contain the soluble proteins extracted from the cytoplasm but leave structural components of the cytoplasm behind. Lysates were centrifuged for 10 min at 4000 rpm at 4°C, supernatants were collected as the "cytosolic” fraction and pellets were collected as the "nonsoluble” or “noncytosolic” fraction containing nuclei and insoluble (e.g. cytoskeleton-associated) proteins. After separation in a 12% PAA gel, proteins were blotted onto PVDF membranes and DDX3 and Grb2 were detected as described in the previous paragraph. Western blot signals were normalized by two approaches: Equal cell numbers were used for preparation of the lysates and densitometric values of antigen-specific bands were then regarded to reflect the amount of the respective protein per cell. These data are shown in the manuscript. In addition, densitometric values were normalized to the amounts of proteins in the respective lane of the blot as determined by densitometric analysis of the Ponceau S staining. These data are shown in the supporting material (S3 and S4 Figs).

Immunofluorescence detection of viral immediate early antigen: Infected cells were fixed with 80% acetone and stained for viral immediate early antigen (pUL122/123) by subsequent incubation with antibody E13 (Biosoft) and Cy3-conjugated goat anti-mouse IgG F(ab)_2_ (Jackson ImmunoResearch). The nuclei were counterstained with DAPI.

Immunofluorescence detection of Grb2: For analysis of the intracellular distribution of Grb2, HFFs grown on 8-well chamber slides (μ-slide, Ibidi) were mock-infected or infected with UL32-EGFP-HCMV [18] at MOIs > 10, fixed with paraformaldehyde at 2 or 6 days and permeabilized with detergent or methanol. Fixed cells were subsequently incubated with a primary antibody against Grb2 (clone 81/GRB2 BD Transduction Laboratories) and a red fluorescent secondary antibody (Cy3-goat anti mouse-Ig Fab'2-fragments; Jackson ImmunoResearch). Cells were then counterstained with DAPI and analyzed with a fluorescence microscope (Zeiss Axio Observer) documenting red immunofluorescence signals, blue DAPI signals and green native fluorescence of EGFP-UL32. To control for nonspecific binding of antibodies, staining with an irrelevant primary mouse antibody (anti-F.VIII, Dako) were included.

Knockdown of gene expression with siRNA: HFFs were transfected with 50nM siRNA targeting Grb2 and DDX3 (M-019220-00 and M-006874-01; Thermo Scientific) using Lipofectamine RNAiMAX transfection reagent (Life Technologies). To control for unspecific effects of siRNA transfection, a pool of non-targeting (NT) siRNAs was included (D-001206-14; Thermo Scientific). As a positive control for an inhibitory effect on HCMV replication, siRNAs directed against the major immediate early (IE) gene region were included that were designed to knockdown UL122 and UL123 transcripts.

## Results and Discussion

### Grb2 and DDX3 are incorporated in HCMV virions in a pp65-dependent fashion

In a proteomic approach, 71 cellular proteins have been reported to be associated with virions [3]. Among these proteins, DDX3 and Grb2 gained our interest because their incorporation into virions was particularly dependent on the viral tegument protein pp65 (pUL83) when we compared the protein composition of a pp65 deletion mutant with wild type virus by two dimensional gel electrophoresis followed by mass spectrometry of individual spots (S2 Fig and S1 Table). Overrepresentation in wild type virions over pp65 deletion virions could result either from pp65-dependent differences in the overall levels of Grb2 and DDX3 in infected cells or from pp65-dependent incorporation during virion maturation. Either of the two explanations is suggestive of a functional relevance of the protein.

**Fig 1:**
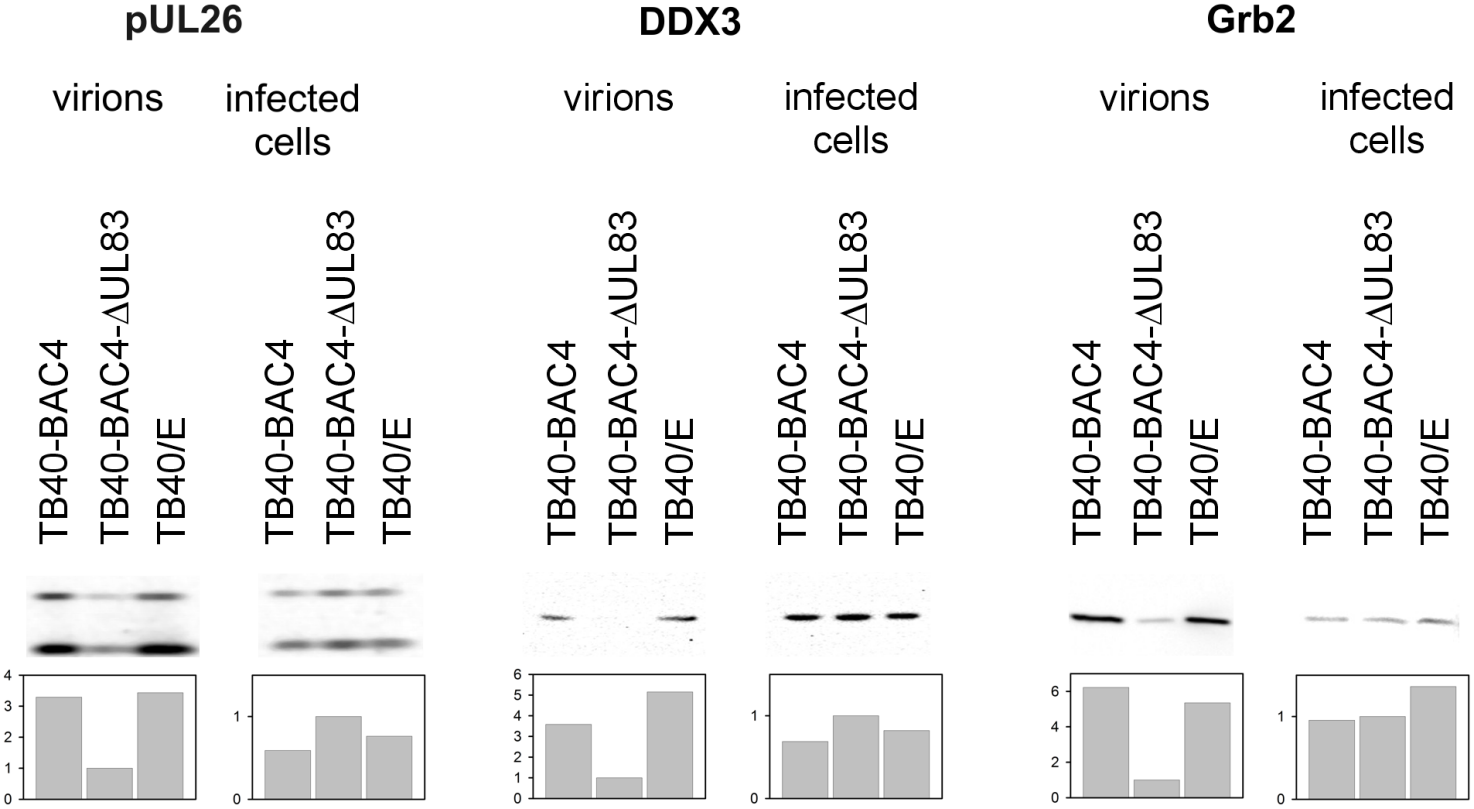
Detection of Grb2 and DDX3 in virions and infected cells. Virions were harvested at 6 d post-infection from cells infected with wild type virus (TB40-BAC4 and TB40/E) or a UL83 deletion mutant, gradient purified and lysed. Cells of the producer cultures were also lysed, and all lysates were analyzed by western blotting for the relative levels of cellular proteins Grb2 and DDX3 and the viral protein pUL26 (two isoforms). For quantification, arbitrary units are given, relative to the content in samples of the UL83-deletion mutant.

Therefore, Grb2 and DDX3 were chosen for further analysis of their impact on HCMV growth. First, we validated the proteomic findings by immunoblotting of independent virion preparations using antibodies against Grb2 and DDX3. The viral UL26-protein known to be incorporated in a pp65-dependent manner [4] served as a positive control. Virions of the pp65 deletion mutant TB40-BAC4-delUL83 were compared to virions of wild type viruses (TB40-BAC4 and the parental strain TB40/E). As no virions can be harvested from mock-infected cells, a mock control was not possible in this experiment. Both the incorporation into virions and the dependence on pp65 were confirmed in this experiment (Fig 1). In addition, comparison with lysates of the infected cells from which virions were harvested showed that pp65-dependent incorporation was not simply due to higher protein levels in cells infected with wild type virus.

### Total levels of Grb2 and DDX3 are increased upon HCMV infection whereas cytosolic Grb2 levels are decreased

Next, the hypothesis that an overall altered subcellular distribution causes the pp65-dependent differences in incorporation of DDX3 and Grb2 was addressed by immunoblotting of cytosolic versus noncytosolic fractions from cells infected for 1–6 days. HFFs were mock-infected or infected with HCMV-TB40-BAC4 and HCMV-TB40-BAC4delUL83 at an MOI of 5 and incubated for 1–6 days. Cells were then trypsinized, lysed with a mild detergent to extract the cytosol from the residual cell fraction, and the levels of Grb2 and DDX3 were determined in the respective subcellular fractions by quantitative western blotting. As equal cell numbers were used for preparation of the various lysates, differences in the Western blot signals reflect relative differences in the amounts of the respective proteins per cell. In addition, the signals were normalized to the global protein content (S4 Fig) with essentially the same results.

**Fig 2:**
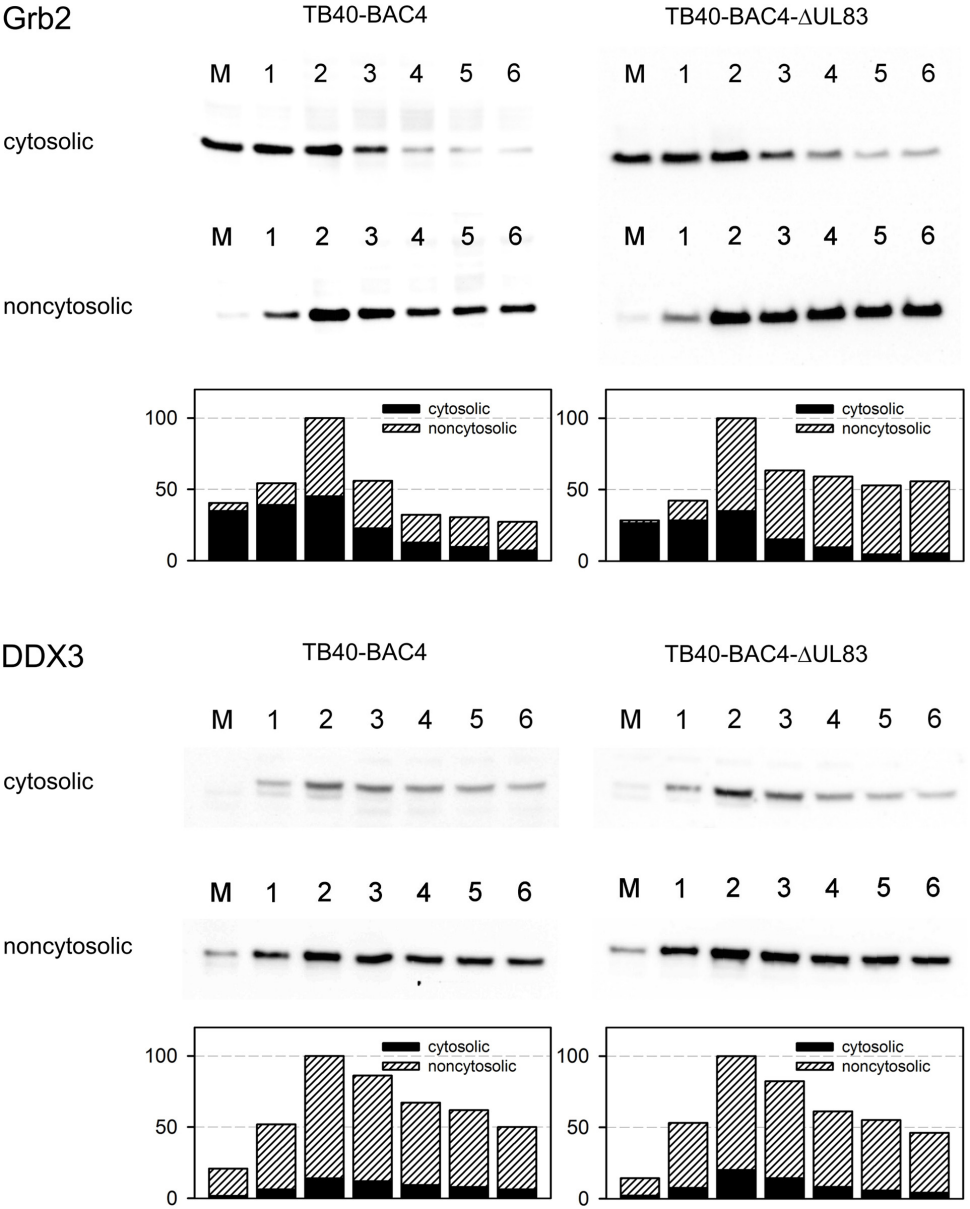
Effect of HCMV infection on levels and distribution of Grb2 and DDX3 within infected cells. Cells were infected with wild type virus (TB40-BAC4) or a UL83-deletion mutant, and lysed with a mild detergent at 1–6 days after infection (d p.i. indicated by lane numbers), yielding a soluble cytosolic fraction and a residual nonsoluble fraction. Both fractions were further lysed with Lämmli buffer and analyzed by western blot for levels of DDX3 and Grb2. Mock-infected cells (M) were included as controls. For quantification, arbitrary units are given, relative to peak levels.

These experiments reproducibly showed a strong upregulation of Grb2 and DDX3 peaking at 2 d post infection (p.i.) and a remarkable shift of Grb2 from the cytosolic fraction to the noncytosolic fraction, which was almost complete at 4 d p.i. both with wild type and with mutant virus (Fig 2). While in mock-infected cells 90% of the signal was found in the cytosolic fraction, this dropped below 50% by 2 d p.i. and further to values below 25% by 6 d p.i. The distribution of DDX3 remained almost unaltered during the replication cycle when compared to mock-infected cells, with 80–90% of the signal found in the noncytosolic fraction and only 10–20% in the cytosolic fraction.

While the blot shown in Fig 2 might give the impression of a slightly higher Grb2 level in mutant-infected cells at 4–6 d p.i. this was not a reproducible finding in repeated assays. On one hand this result failed to provide an explanation for the pp65-dependent incorporation of Grb2 and DDX3 into virions, on the other hand the remarkable upregulation (DDX3 and Grb2) and redistribution (Grb2) upon HCMV infection nevertheless increased our interest in the question of whether these proteins may have a functional role during viral replication.

The shift of Grb2 from the cytosolic to the noncytosolic fraction could either indicate a translocation towards the nucleus or a sequestration in the cytoplasm in a nonsoluble form. To resolve this we stained Grb2 by immunofluorescence in noninfected cells and cells at 2 d p.i. and 6 d p.i., which were the time points when induction or redistribution were maximal, respectively. HFFs were mock-infected or infected with UL32-EGFP-HCMV-TB40 [18] at MOIs > 10, fixed with paraformaldehyde at 2 or 6 days, permeabilized and stained for Grb2 by indirect immunofluorescence, including irrelevant mouse antibodies (anti-F.VIII, Dako) as controls. Stained samples were then analyzed with regard to changes in the overall level of Grb2 upon infection and its intracellular distribution. Furthermore, the staining procedure preserved the native fluorescence of EGFP and therefore allowed to compare the localization of the viral structural protein pUL32 with the localization of Grb2.

**Fig 3:**
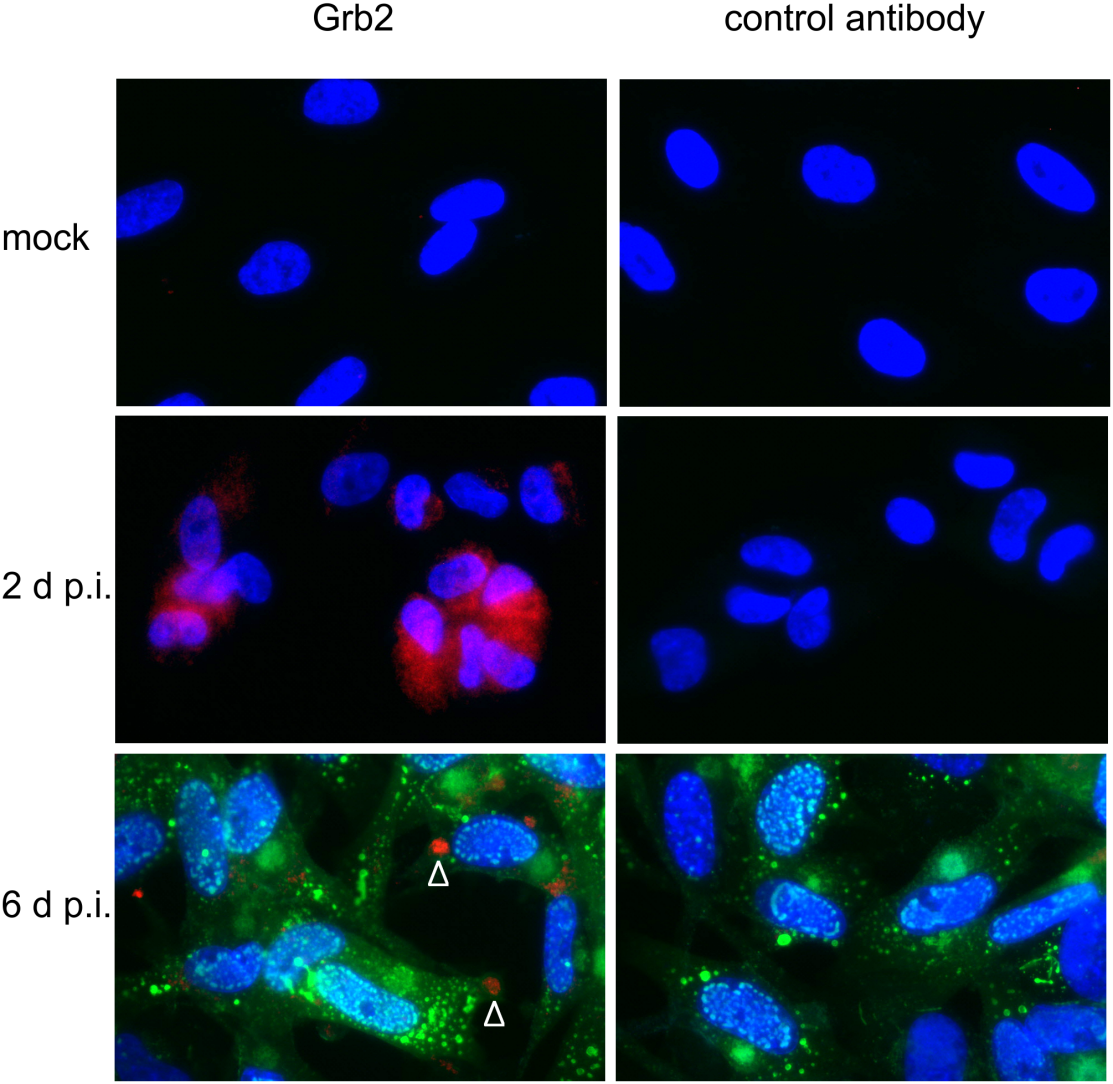
Distribution of Grb2 in infected cells. Cells were infected with virus carrying a green fluorescent label at the capsid-associated tegument protein pUL32 (UL32-EGFP-HCMV-TB40). At 2 d p.i. and 6 d p.i. cells were fixed, stained by immunofluorescence with a Grb2-specific antibody or a F.VIII-specific control antibody, and counterstained with DAPI. Merged images taken at a 630fold magnification are shown combining red Grb2 immunofluorescence (Zeiss filter set 14), blue nuclear DAPI signals (Zeiss filter set 01) and green native fluorescence (Zeiss filter set 09) of the viral EGFP-tagged tegument protein pUL32.

Repeated analyses consistently showed (i) the upregulation of Grb2 in infected cells as compared to mock-infected cells, with (ii) a localization in the cytoplasm at 2 d p.i. and (iii) a redistribution of cytoplasmic Grb2 towards circumscribed regions in the cell periphery at 6 d p.i. (Fig 3; for example see arrowheads).

The fact that IF staining did not yield specific Grb2 signals in mock-infected cells whereas Western blotting did, indicates that IF staining were less sensitive. At least Grb2 staining were sensitive enough to detect the increased levels in infected cells. In contrast, none of our DDX3-specific antibodies yielded reliable signals, i.e. signals above the levels obtained with irrelevant antibodies. Hence, the expression of DDX3 in infected cells could not be addressed by IF staining.

### Knockdown of Grb2 and DDX3 reduces the efficiency of viral spread

**Fig 4:**
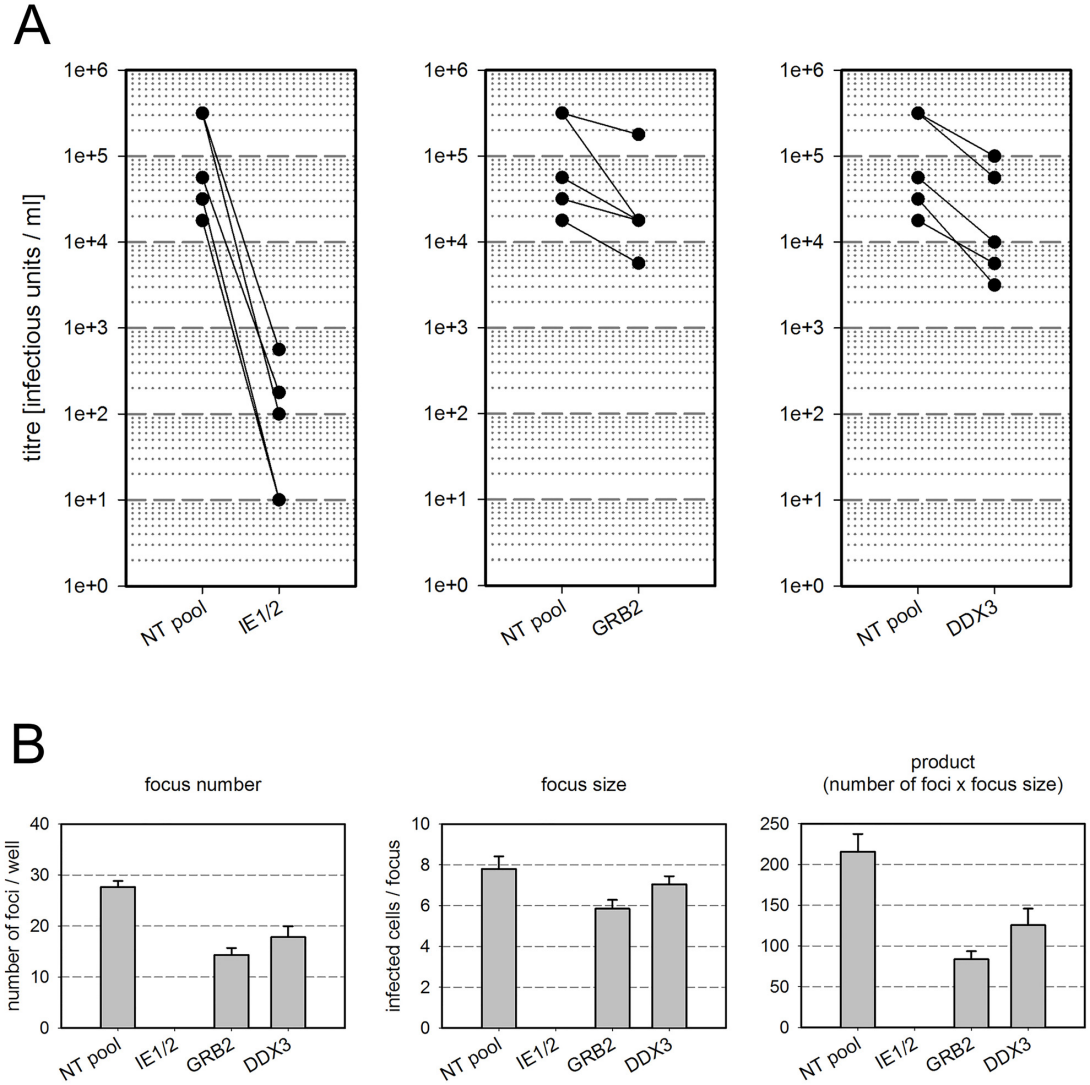
Effect of Grb2 and DDX3 on viral growth. Human foreskin fibroblasts were treated with siRNA against the viral immediate early genes UL122/123 (IE1/2, positive control), the cellular gene Grb2, the cellular gene DDX3 or a non-targeting siRNA (negative control). Two days after siRNA treatment, cells were infected with HCMV strain TB40/E at an MOI of 1(A) or 0.01 (B). A: The titer of infectious progeny virus in cell free supernatants of cells infected at an MOI of 1 was determined at 5 d postinfection. Each matched pair of values represents an independent experiment. Knockdown of both Grb2 and DDX3 reduced virus progeny production. B: Focal growth in cultures infected at an MOI of 0.01 was determined by staining cells for the viral immediate early antigens and counting the number of foci per well and the number of infected cells per focus (focus size). The total effect on focal growth is represented by the product of focus number and focus size.

To test whether Grb2 and DDX3 contribute to HCMV replication, an siRNA-based knockdown approach was applied. HFFs were transfected with siRNAs targeting Grb2 or DDX3, and 2 d after transfection cells were infected with HCMV strain TB40/E at an MOI of 1. Non-targeting (NT) siRNA and siRNA directed against the major immediate early (IE) gene region were included as negative or positive controls, respectively. The efficiency of the knockdown was proven by immunoblotting performed in parallel and the viability of the respective cell cultures was tested by CellTiter-Blue assays (S5 Fig panels A, B). At 5 d p.i., supernatants were harvested and the infectivity of cell-free infectious progeny virus was determined by limiting dilution assays (TCID50) using fibroblasts as indicator cells and immunofluorescence staining for viral IE antigens as a readout. Virus progeny in the supernatant of infected cells was significantly reduced in 5 experiments by knockdown of either Grb2 (0.38 of NT control; p = 0.02) or DDX3 (0.18 of NT control; p = 0.001) (Fig 4A). As a positive control, knockdown of major immediate early-RNAs almost completely blocked production of virus progeny (0.001 of NT control; p < 0.0001).

To confirm a possible contribution of Grb2 and DDX3 to the replicative success of the virus by an independent readout, we investigated whether spread to neighboring cells and focus formation by HCMV strain TB40/E is impaired upon silencing of GRB2 or DDX3. For this, we infected siRNA-transfected cells at an MOI of 0.01, incubated the cultures for 4 d to allow for focus formation and visualized infectious foci by immunofluorescence for IE antigens at 4 d p.i. Both the number of foci (defined as sites with ≥ 3 infected cells) and focus size (number of IE antigen-positive nuclei per focus) were evaluated. The product of focus size and focus number was calculated to reflect the overall viral spreading capacity. The initial infection rate as determined in a replica assay at 1 dp.i. was 1% irrespective of whether Grb2, DDX3 and NT RNA pools were applied (S5 Fig panel C), arguing against an effect on initial events of infection by knockdown of Grb2 or DDX3. In contrast, IE-specific siRNAs expectedly suppressed IE antigen expression to undetectable levels under these conditions. Overall focal growth, represented by the product of focus number and focus size, was significantly reduced in three experiments with knockdown of either Grb2 or DDX3. This was in part due to the reduction of the number of foci and in part to the size of the remaining foci (one representative experiment with 6 replica wells is shown in Fig 4B; Grb2 = 0.39 of NT control; p < 0.01; DDX3 = 0.58 of NT control; p < 0.05). Knockdown of major immediate early-RNAs had the expected strong inhibitory effect (p < 0.01). When analyzed individually, focus numbers were significantly reduced with siRNA against IE1/2 (p < 0.01), Grb2 (p < 0.01) and DDX3 (p < 0.05) while reduction of focus size was only significant with siRNAs against IE1/2 (p < 0.0001) and Grb2 (p < 0.05).

**Fig 5:**
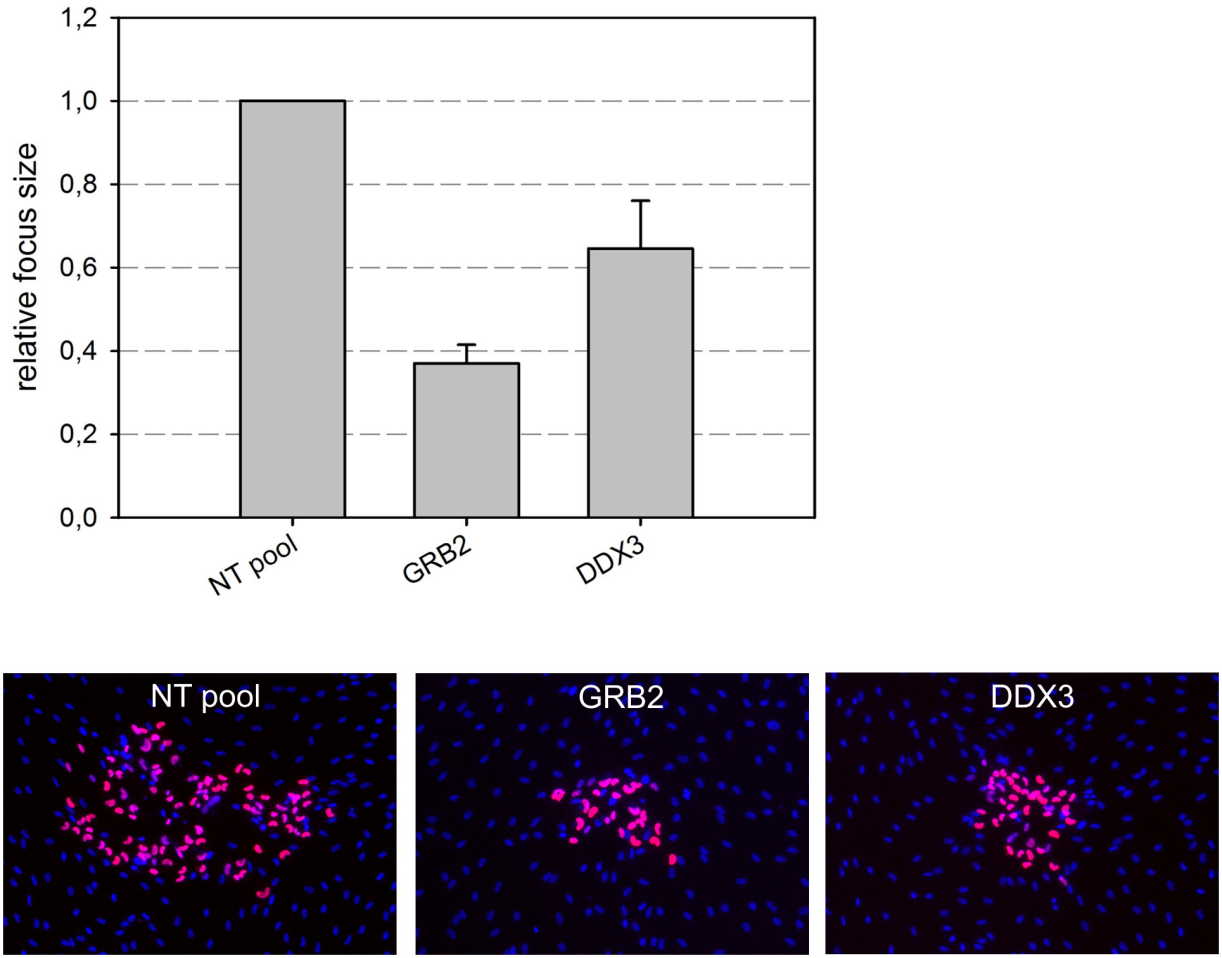
Effect of Grb2 and DDX3 on focal growth of clinical HCMV isolates. Human foreskin fibroblasts were treated with siRNA against the cellular gene Grb2, the cellular gene DDX3 or a non-targeting siRNA (negative control) one day prior to coculture with cells that were infected with clinical HCMV isolates. Seven days after coculture, cells were fixed, stained for viral immediate early antigens, and focus size was determined as the number of infected cells per focus. The mean relative focus size for Grb2- and DDX3-specific siRNAs as compared to non-targeting siRNA control with four different isolates is shown (upper panel). Representative examples of foci are shown in the lower panel.

Finally, we tested whether knockdown of Grb2 and DDX3 also affected the growth of recent clinical isolates. As recent clinical isolates grow cell-associated and do not release measurable infectivity into the supernatant, only focus-growth assays could be applied. HFFs were transfected with the respective siRNAs, cocultured with isolate-infected cells at 24 h after transfection, and incubated for 7 d to allow for focus formation. Cultures were then fixed, viral IE antigens were detected with indirect immunofluorescence and the focus size was determined as described before [23]. Like with HCMV-TB40/E, focal growth of clinical HCMV isolates from four different patients was restricted by knockdown of Grb2 (0.37 of NT control; p < 0.01) or DDX3 (0.65 of NT control; p = 0.026) (Fig 5).

In conclusion, we identified two cellular proteins, Grb2 and DDX3 that are upregulated in infected cells, are incorporated into virions, and support viral spread. Both proteins have been found in virions in a previous analysis of virions of strain AD169 [3] while their increased incorporation in wild type-virus versus pp65-deletion mutants is a novel finding. The reason why these proteins were not detected in a recent comparison of virions from AD169 and AD169derived pp65 deletion viruses [4] is unclear, considering their abundance in the previous analysis of AD169 virions [3]. Importantly, our confirmation of the proteomic data by independent Western blot analyses corroborated the notion that Grb2 and DDX3 are actually incorporated into virions. Generally, proteomic analyses should not be regarded comprehensive, as differences in the protocols for generation of the protein lysates may account for the exclusion of different sets of proteins. This may also explain why only two of the viral-encoded virion proteins reported to depend on pp65 [4, 24] were above the threshold of pp65-specific incorporation in our proteomic screening.

DDX3 is a multifunctional protein, found both in the nucleus and the cytoplasm, contributing to RNA splicing, mRNA export, transcriptional regulation, translational regulation, RNA decay and ribosome biogenesis via its RNA-binding and RNA-helicase activity [25]. In virus infections, opposing effects of DDX3 were reported with different viruses. On one hand, it was suggested to be involved in the induction of anti-viral mediators [6, 26–28]. On the other hand, HIV and HCV require DDX3 for their replication [29–32]. In line with the latter, we found DDX3 upregulated upon infection with a maximum expression at 2 d p.i., concordant with recent publications [11, 33], and viral replication restricted under conditions of DDX3 knockdown. The effect of the DDX3 knockdown on the release of infectious progeny was slightly stronger when compared to Grb2 while the effect on focal growth was weaker.

Grb2 is physiologically associated with growth factor receptors. It is noteworthy that growth factor receptors have been reported to contribute to HCMV entry [34–36], which is, however, controversial as evidence against a contribution to viral entry has also been published [37–39]. We found no effect of Grb2 knockdown on the initial infection rate (S5 Fig panel C of the supporting material), arguing against a contribution during entry. This is in line with our finding of Grb2 peaking at 2 d p.i., a phase when late structural proteins start being expressed. Both facts together suggests a contribution of Grb2 later during the replication cycle. The same expression pattern was found recently in a proteomic approach [11]. Consistent with this expression pattern, Grb2 knockdown restricted viral spread. Both titers of infectious virus released by infected cells and the efficiency of focal growth were 2-3fold reduced when Grb2 expression was reduced by small interfering RNAs. Importantly, Grb2 knockdown also restricted the cell-associated focal growth of a recent clinical isolate, further emphasizing the relevance of this cellular protein for successful HCMV replication. Regarding the underlying mechanisms, we can only speculate at the moment, and both indirect effects on the cellular state via the involvement of Grb2 in the growth factor signaling cascade and direct effects via interaction with viral proteins are possible. Our immunofluorescence data do not support the idea that Grb2 becomes associated with the viral assembly compartment in the late stage of infection, favoring a more indirect role in HCMV replication. The focal accumulations of Grb2 in late stage infected cells most probably reflect the progressive shift of this protein towards an insoluble compartment during the replication cycle, while the exact nature of these accumulations remains to be identified. The role of the viral protein pp65 is ambiguous: on one hand, usage of the pp65 deletion mutant indicated the specific incorporation of Grb2 and DDX3 into HCMV virions. On the other hand, deletion of pp65 did not notably alter levels and distribution of these proteins in infected cells, arguing against a redirection of these proteins by pp65 to sites of viral replication. This is further supported by the fact the Grb2 was not particularly colocalized with the capsid-associated tegument protein pp150. Also, the fact that deletion of pp65 in the background of HCMV strains AD169 [40] and TB40/E [24] has no effect on viral replication in HFFs (see also S1 Fig panel C of the supporting material) whereas knockdown of Grb2 and DDX3 has an inhibitory effect, argues against an essential contribution of pp65 to the proviral effects of these cellular proteins.

Questions to be addressed in the future are how HCMV infection increases the expression of Grb2 and DDX3 and how, in turn, these cellular proteins promote viral spread. It will be important to discriminate whether Grb2 and DDX3 support viral replication simply via an increase of their physiological functions upon upregulation or via a more specific interaction with viral proteins during virus maturation. If the latter applies, this could provide a target for specific antiviral strategies to reduce viral growth in infected cells while preserving the physiological functions of Grb2 and DDX3 in uninfected cells.

## Supporting Information

S1 Fig Characterization of the UL83 deletion mutant. (A) Virus particles were separated by ultracentrifugation through a density/viscosity gradient (tartrate/glycerol). HCMV-TB40-BAC4delUL83 showed a virion band similar to wild type viruses, but lacked the typical unsharp band originating from dense body in the lower half of the gradient. This is consistent with the lack of pUL83, which is the major constituent of dense bodies. (B) When virions bands were lysed and analyzed for the presence of pUL83 by western blotting, mutant virions lacked a specific band as compared to wild type virions. In contrast, levels of a control tegument protein (pUL32) were similar with both virion preparations. (C) Growth properties of mutant and wild type virus were analyzed by single step growth curves. Fibroblast cultures were infected at infection multiplicities of 3 infectious units / cell and supernatants were collected daily between 1 and 8 days postinfection (p.i.). Virus progeny in the supernatants was quantitatively determined by limiting dilution assays (TCID50 = tissue culture infective dose 50%) using fibroblasts as indicator cells and detection of viral immediate early antigens as a readout. (D) Supernatants of mutant and wild type virus were analyzed regarding loss of infectivity over time by repeated quantification of the infectivity at different time points after the harvest. Apparently, the viruses did not differ regarding the kinetics of decay of their biological activity.
(TIF)

S2 Fig Proteomic in-gel comparison of virions from UL83-deletion mutant and wt virus. Protein lysates of gradient purified virions from TB40/E and the TB40/E-derived UL83-deletion mutant were labelled with Cy3 and Cy5, respectively. For an internal standard, lysates of both viruses were pooled and labelled with Cy2. A mixture of all three samples was run in 2D gel electrophoresis. (A) Indication of differentially represented spots as detected by Decyder analysis. (B) Interstrain comparison of spots with significant differences in the Decyder analysis. a = UL83 deletion virus, b = wild type virus. (C) Three-dimensional presentation of spots 3–7 (UL99) and spots 9–10 (Grb2) demonstrates the strong differences in expression of these proteins. a = UL83 deletion virus, b = wild type virus.
(TIF)

S3 Fig Examples of Ponceau S stained blots as used for densitometric analyses. Blots were stained with Ponceau S and the protein content of each lane was evaluated by densitometric analysis to serve as a reference value for normalization of western blot signals. Examples of blots used for the generation of figs 1 and 2 of the manuscript are shown here.
(TIF)

S4 Fig Effect of HCMV infection on levels and distribution of Grb2 and DDX3 within infected cells. Cells were infected with wild type virus (TB40-BAC4) or a UL83-deletion mutant, and lysed with a mild detergent at 1–6 days after infection (d p.i. indicated by lane numbers), yielding a soluble cytosolic fraction and a residual nonsoluble fraction. Both fractions were further lysed with Lämmli buffer and analyzed by western blot for levels of DDX3 and Grb2. Western blot signals were analyzed by densitometry and normalized to the global amount of protein in the respective lane as determined by densitometric analysis of the Ponceau S staining. Mock-infected cells (M) were included as controls. For quantification, arbitrary units are given, relative to peak levels.
(TIF)

S5 Fig Effect of siRNAs on protein expression, cell viability and infection rates. (A) HFFs were transfected with 50 nM siRNA targeting viral immediate early genes Ul122/123, cellular genes Grb2 and DDX3 or a pool of non-targeting (NT) siRNAs using Lipofectamine RNAiMAX transfection reagent (Life Technologies) and infected with HCMV strain TB40/E 48 hours after transfection at an approximate MOI of 1. At 5 d postinfection (p.i.) virus progeny was harvested. To control for the efficiency of the siRNA-mediated knockdown, cells were lysed and analyzed by western blotting under denaturing reducing conditions. (B) At 2 days after transfection of the respective siRNAs, cell viability was analyzed by measuring the metabolic activity of the cell cultures with the CellTiter-Blue Cell Viability Assay (Promega), using the indicator dye resazurin which is reduced to the highly fluorescent compound resorufin by metabolically active viable cells. (C) To test the effect of the respective siRNAs on infection efficiency, cells were infected at 2 days after transfection at an infection multiplicity of 0.01 infectious units (IU) per cell. At 1 day p.i., cells were stained for viral immediate early antigens by indirect immunofluorescence.
(TIF)

S1 Table Identification of pp65-dependent virion proteins. The protein patterns of delUL83-mutant and wild type virions were compared in a DIGE analysis. Virions were harvested from infected HFF and purified in glycerol-tartrate gradients. After lysis with 1% SDS, proteins were purified using the 2D clean up kit (GE Healthcare Bio-Sciences; "GE Healthcare"). Protein samples of mutant and wild type virions were resuspended in DIGE buffer (7 M urea, 2 M thiourea, 4% CHAPS, 30 mM Tris) at 2 mg/ml and labelled with Cy3 and Cy5 and vice versa (GE Healthcare). Internal pooled standard was prepared from equal amounts of each sample and labelled with Cy2. For each gel, 50 μg aliquots of Cy2-, Cy3- and Cy5-labelled samples were pooled and the final volume was adjusted to 460 μl with rehydration buffer (7 M urea, 2 M thiourea, 2% CHAPS, 1% DTT, 2% Pharmalyte, pH 3–10) and loaded on pH 3–10 gel strips (Immobiline DryStrips, GE Healthcare). Following overnight rehydration, isoelectric focusing was done in an IPGphor focusing unit for a total of 65.5 kVh. Gel strips were subsequently equilibrated twice for 15 min in equilibration buffer containing 6 M Urea, 30% glycerol (Sigma), 2% w/v SDS (Serva) and a trace of bromophenol blue, supplemented with 5 mg/ml DTT (Biorad) or 45 mg/ml iodacetamide (Fluka), respectively. Proteins were then separated in 12.5% SDS-PAGE gels using the *DALTsix* electrophoresis system (GE Healthcare). Gels were scanned with a Typhoon 9410 Variable Imager (GE Healthcare) and image analysis was performed with Decyder Differential Analysis Software version v7.5 (GE Healthcare). Parameters for the spot detection algorithm were set to “slope” 3 and “estimated number of spots” = 2000. For statistical analysis, the Student’s *t-*test *p* value was set to 0.05 with an average ratio of spots greater than 1.5 times intensity. Virion samples of both viruses from three independent gradient purifications were compared in three DIGE gels. Deletion of UL83 had a dramatic impact on the protein composition of the virion. Reproducible differential spots were detected with the Decyder v.7.5 software (see S2 Fig). Fluorescence gels were subsequently stained by mass-spectrometry-compatible silver staining for visualization of spots and 5 of the differential spots were identified directly from these DIGE gels by ESI-MS/MS. Six additional differential spots were identified indirectly by ESI-MS/MS of respective spots from a Bac4 virion 2-D coomassie-stained gel. All spots were identified by searching peptides in the human and viral NCBI sequence databases using the Mascot search engine (Matrix Science).
(PDF)

## References

[1] Gibson W. Structure and formation of the cytomegalovirus virion. Curr Top Microbiol Immunol. 2008;325:187–204. Epub 2008/07/22. . 18637507 10.1007/978-3-540-77349-8_11

[2] Baldick CJ Jr., Shenk T. Proteins associated with purified human cytomegalovirus particles. J Virol. 1996;70(9):6097–105. Epub 1996/09/01. 8709233 10.1128/jvi.70.9.6097-6105.1996 PMC190631

[3] Varnum SM, Streblow DN, Monroe ME, Smith P, Auberry KJ, Pasa-Tolic L, et al Identification of proteins in human cytomegalovirus (HCMV) particles: the HCMV proteome. J Virol. 2004;78(20):10960–6. Epub 2004/09/29. 10.1128/jvi.78.20.10960-10966.2004 15452216 PMC521840

[4] Reyda S, Tenzer S, Navarro P, Gebauer W, Saur M, Krauter S, et al The tegument protein pp65 of human cytomegalovirus acts as an optional scaffold protein that optimizes protein uploading into viral particles. J Virol. 2014;88(17):9633–46. Epub 2014/06/13. 10.1128/JVI.01415-14 JVI.01415-14 [pii]. 24920816 PMC4136338

[5] Allal C, Buisson-Brenac C, Marion V, Claudel-Renard C, Faraut T, Dal Monte P, et al Human cytomegalovirus carries a cell-derived phospholipase A2 required for infectivity. J Virol. 2004;78(14):7717–26. Epub 2004/06/29. 10.1128/jvi.78.14.7717-7726.2004 15220446 PMC434095

[6] Wang H, Kim S, Ryu WS. DDX3 DEAD-Box RNA helicase inhibits hepatitis B virus reverse transcription by incorporation into nucleocapsids. J Virol. 2009;83(11):5815–24. Epub 2009/03/20. 10.1128/jvi.00011-09 19297497 PMC2681949

[7] Ott DE. Cellular proteins detected in HIV-1. Rev Med Virol. 2008;18(3):159–75. Epub 2008/02/12. 10.1002/rmv.570 . 18265424

[8] Stegen C, Yakova Y, Henaff D, Nadjar J, Duron J, Lippe R. Analysis of virion-incorporated host proteins required for herpes simplex virus type 1 infection through a RNA interference screen. PLoS One. 2013;8(1):e53276 Epub 2013/01/10. 10.1371/journal.pone.0053276 PONE-D-12-31532 [pii]. 23301054 PMC3536771

[9] Child SJ, Jarrahian S, Harper VM, Geballe AP. Complementation of vaccinia virus lacking the double-stranded RNA-binding protein gene E3L by human cytomegalovirus. J Virol. 2002;76(10):4912–8. Epub 2002/04/23. 11967308 10.1128/JVI.76.10.4912-4918.2002 PMC136161

[10] Browne EP, Wing B, Coleman D, Shenk T. Altered cellular mRNA levels in human cytomegalovirus-infected fibroblasts: viral block to the accumulation of antiviral mRNAs. J Virol. 2001;75(24):12319–30. Epub 2001/11/17. 10.1128/jvi.75.24.12319-12330.2001 11711622 PMC116128

[11] Weekes MP, Tomasec P, Huttlin EL, Fielding CA, Nusinow D, Stanton RJ, et al Quantitative temporal viromics: an approach to investigate host-pathogen interaction. Cell. 2014;157(6):1460–72. 10.1016/j.cell.2014.04.028 24906157 PMC4048463

[12] Landais I, Nelson JA. Functional genomics approaches to understand cytomegalovirus replication, latency and pathogenesis. Curr Opin Virol. 2013;3(4):408–15. Epub 2013/07/03. 10.1016/j.coviro.2013.06.002 S1879-6257(13)00096-5 [pii]. 23816389 PMC3748260

[13] Belov AA, Mohammadi M. Grb2, a double-edged sword of receptor tyrosine kinase signaling. Science signaling. 2012;5(249):pe49 10.1126/scisignal.2003576 23131845 PMC3668340

[14] Fortian A, Sorkin A. Live-cell fluorescence imaging reveals high stoichiometry of Grb2 binding to the EGF receptor sustained during endocytosis. Journal of cell science. 2014;127(Pt 2):432–44. 10.1242/jcs.137786 24259669 PMC3889400

[15] Schroder M. Human DEAD-box protein 3 has multiple functions in gene regulation and cell cycle control and is a prime target for viral manipulation. Biochemical pharmacology. 2010;79(3):297–306. 10.1016/j.bcp.2009.08.032 . 19782656

[16] Sinzger C, Schmidt K, Knapp J, Kahl M, Beck R, Waldman J, et al Modification of human cytomegalovirus tropism through propagation in vitro is associated with changes in the viral genome. The Journal of general virology. 1999;80 (Pt 11):2867–77. Epub 1999/12/02. . 10580048 10.1099/0022-1317-80-11-2867

[17] Sinzger C, Hahn G, Digel M, Katona R, Sampaio KL, Messerle M, et al Cloning and sequencing of a highly productive, endotheliotropic virus strain derived from human cytomegalovirus TB40/E. The Journal of general virology. 2008;89(Pt 2):359–68. Epub 2008/01/17. 89/2/359 [pii] 10.1099/vir.0.83286-0 . 18198366

[18] Laib Sampaio K, Cavignac Y, Stierhof YD, Sinzger C. Human cytomegalovirus labeled with green fluorescent protein for live analysis of intracellular particle movements. J Virol. 2005;79(5):2754–67. Epub 2005/02/15. 79/5/2754 [pii] 10.1128/JVI.79.5.2754-2767.2005 15708994 PMC548422

[19] Hierholzer JC, Killington RA. 2—Virus isolation and quantitation In: Kangro BWJMO, editor. Virology Methods Manual. London: Academic Press; 1996 p. 25–46.

[20] Talbot P, Almeida JD. Human cytomegalovirus: purification of enveloped virions and dense bodies. The Journal of general virology. 1977;36(2):345–9. Epub 1977/08/01. . 197204 10.1099/0022-1317-36-2-345

[21] Stamminger T, Gstaiger M, Weinzierl K, Lorz K, Winkler M, Schaffner W. Open reading frame UL26 of human cytomegalovirus encodes a novel tegument protein that contains a strong transcriptional activation domain. J Virol. 2002;76(10):4836–47. . 11967300 10.1128/JVI.76.10.4836-4847.2002 PMC136153

[22] Angus AG, Dalrymple D, Boulant S, McGivern DR, Clayton RF, Scott MJ, et al Requirement of cellular DDX3 for hepatitis C virus replication is unrelated to its interaction with the viral core protein. The Journal of general virology. 2010;91(Pt 1):122–32. 10.1099/vir.0.015909-0 19793905 PMC2885062

[23] Sinzger C, Knapp J, Plachter B, Schmidt K, Jahn G. Quantification of replication of clinical cytomegalovirus isolates in cultured endothelial cells and fibroblasts by a focus expansion assay. J Virol Methods. 1997;63(1–2):103–12. Epub 1997/01/01. . 9015280 10.1016/s0166-0934(97)02082-x

[24] Chevillotte M, Landwehr S, Linta L, Frascaroli G, Luske A, Buser C, et al Major tegument protein pp65 of human cytomegalovirus is required for the incorporation of pUL69 and pUL97 into the virus particle and for viral growth in macrophages. J Virol. 2009;83(6):2480–90. 10.1128/JVI.01818-08 19116255 PMC2648260

[25] Schroder M. Viruses and the human DEAD-box helicase DDX3: inhibition or exploitation? Biochem Soc Trans. 2011;39(2):679–83. Epub 2011/03/25. 10.1042/bst0390679 . 21428961

[26] Fullam A, Schroder M. DExD/H-box RNA helicases as mediators of anti-viral innate immunity and essential host factors for viral replication. Biochim Biophys Acta. 2013;1829(8):854–65. Epub 2013/04/10. 10.1016/j.bbagrm.2013.03.012 . 23567047 PMC7157912

[27] Schroder M, Baran M, Bowie AG. Viral targeting of DEAD box protein 3 reveals its role in TBK1/IKKepsilon-mediated IRF activation. EMBO J. 2008;27(15):2147–57. Epub 2008/07/19. 10.1038/emboj.2008.143 18636090 PMC2516890

[28] Soulat D, Burckstummer T, Westermayer S, Goncalves A, Bauch A, Stefanovic A, et al The DEAD-box helicase DDX3X is a critical component of the TANK-binding kinase 1-dependent innate immune response. EMBO J. 2008;27(15):2135–46. Epub 2008/06/28. 10.1038/emboj.2008.126 18583960 PMC2453059

[29] Owsianka AM, Patel AH. Hepatitis C virus core protein interacts with a human DEAD box protein DDX3. Virology. 1999;257(2):330–40. . 10329544 10.1006/viro.1999.9659

[30] Yedavalli VS, Neuveut C, Chi YH, Kleiman L, Jeang KT. Requirement of DDX3 DEAD box RNA helicase for HIV-1 Rev-RRE export function. Cell. 2004;119(3):381–92. . 15507209 10.1016/j.cell.2004.09.029

[31] Ariumi Y, Kuroki M, Abe K, Dansako H, Ikeda M, Wakita T, et al DDX3 DEAD-box RNA helicase is required for hepatitis C virus RNA replication. J Virol. 2007;81(24):13922–6. Epub 2007/09/15. 10.1128/jvi.01517-07 17855521 PMC2168844

[32] Randall G, Panis M, Cooper JD, Tellinghuisen TL, Sukhodolets KE, Pfeffer S, et al Cellular cofactors affecting hepatitis C virus infection and replication. Proc Natl Acad Sci U S A. 2007;104(31):12884–9. Epub 2007/07/10. 10.1073/pnas.0704894104 17616579 PMC1937561

[33] Reyda S, Buscher N, Tenzer S, Plachter B. Proteomic analyses of human cytomegalovirus strain AD169 derivatives reveal highly conserved patterns of viral and cellular proteins in infected fibroblasts. Viruses. 2014;6(1):172–88. Epub 2014/01/10. 10.3390/v6010172 24402306 PMC3917437

[34] Chan G, Nogalski MT, Yurochko AD. Activation of EGFR on monocytes is required for human cytomegalovirus entry and mediates cellular motility. Proc Natl Acad Sci USA. 2009;106(52):22369–74. 10.1073/pnas.0908787106 20018733 PMC2799688

[35] Wang X, Huong S-M, Chiu ML, Raab-Traub N, Huang E-S. Epidermal growth factor receptor is a cellular receptor for human cytomegalovirus. Nature. 2003;424(6947):456–61. 12879076 10.1038/nature01818

[36] Soroceanu L, Akhavan A, Cobbs CS. Platelet-derived growth factor-alpha receptor activation is required for human cytomegalovirus infection. Nature. 2008;455(7211):391–5. Epub 2008/08/15. nature07209 [pii] 10.1038/nature07209 . 18701889

[37] Cobbs CS, Soroceanu L, Denham S, Zhang W, Britt WJ, Pieper R, et al Human cytomegalovirus induces cellular tyrosine kinase signaling and promotes glioma cell invasiveness. J Neurooncol. 2007;85(3):271–80. Epub 2007/06/26. 10.1007/s11060-007-9423-2 . 17589804

[38] Isaacson MK, Feire AL, Compton T. Epidermal growth factor receptor is not required for human cytomegalovirus entry or signaling. J Virol. 2007;81(12):6241–7. 17428848 10.1128/JVI.00169-07 PMC1900073

[39] Vanarsdall AL, Wisner TW, Lei H, Kazlauskas A, Johnson DC. PDGF receptor-alpha does not promote HCMV entry into epithelial and endothelial cells but increased quantities stimulate entry by an abnormal pathway. PLoS Pathog. 2012;8(9):e1002905 Epub 2012/10/03. 10.1371/journal.ppat.1002905 23028311 PMC3441672

[40] Schmolke S, Kern HF, Drescher P, Jahn G, Plachter B. The dominant phosphoprotein pp65 (UL83) of human cytomegalovirus is dispensable for growth in cell culture. J Virol. 1995;69(10):5959–68. . 7666500 10.1128/jvi.69.10.5959-5968.1995 PMC189491

