# Supplementary figures and images for: The Cellular Proteins Grb2 and DDX3 Are Increased upon Human Cytomegalovirus Infection and Act in a Proviral Fashion
Source: PLoS One. 2015 Jun 29;10(6):e0131614. doi: 10.1371/journal.pone.0131614 (PMC4509573; doi:10.1371/journal.pone.0131614)

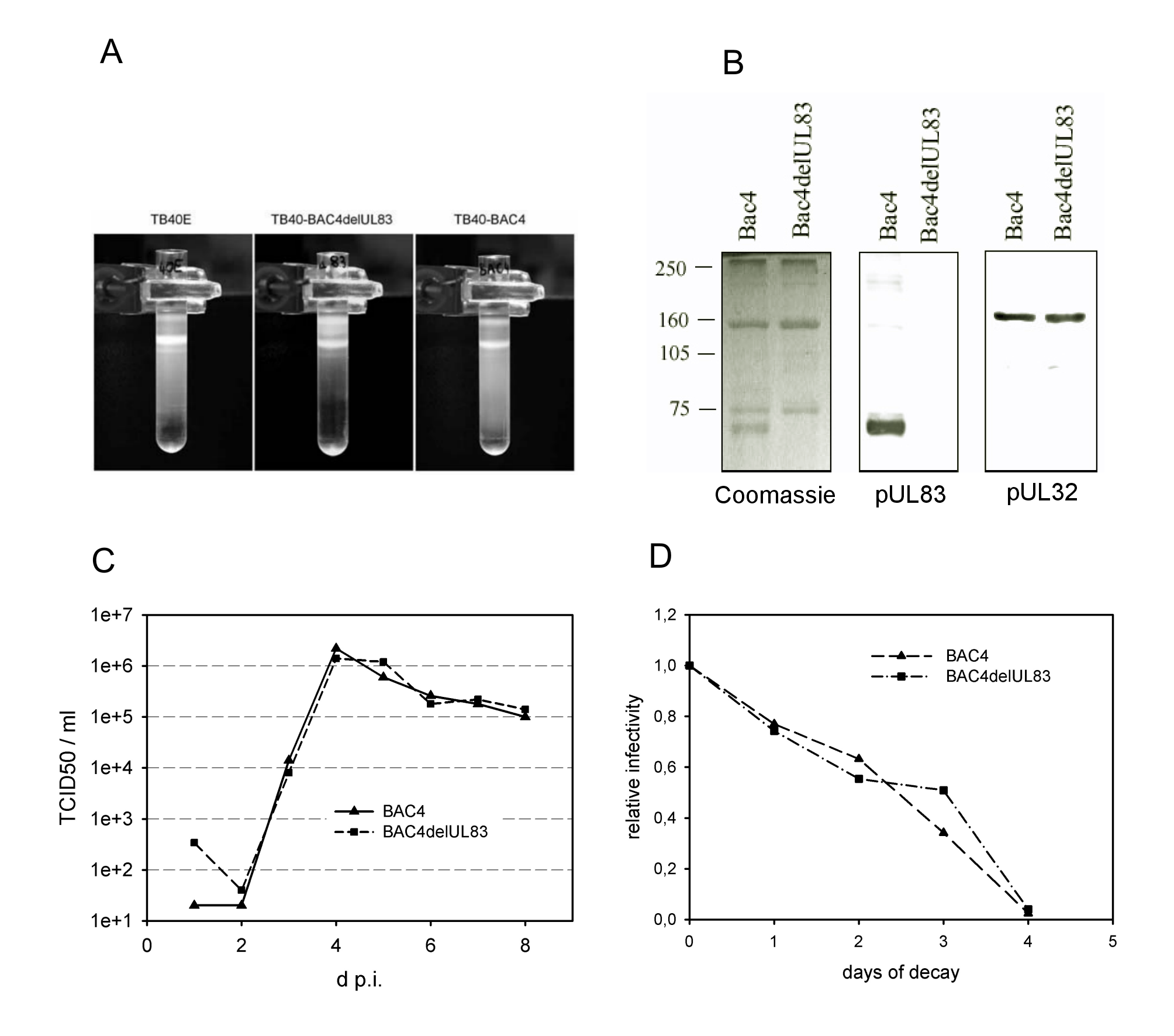

Supplement: S1 Fig — (A) Virus particles were separated by ultracentrifugation through a density/viscosity gradient (tartrate/glycerol). HCMV-TB40-BAC4delUL83 showed a virion band similar to wild type viruses, but lacked the typical unsharp band originating from dense body in the lower half of the gradient. This is consistent with the lack of pUL83, which is the major constituent of dense bodies. (B) When virions bands were lysed and analyzed for the presence of pUL83 by western blotting, mutant virions lacked a specific band as compared to wild type virions. In contrast, levels of a control tegument protein (pUL32) were similar with both virion preparations. (C) Growth properties of mutant and wild type virus were analyzed by single step growth curves. Fibroblast cultures were infected at infection multiplicities of 3 infectious units / cell and supernatants were collected daily between 1 and 8 days postinfection (p.i.). Virus progeny in the supernatants was quantitatively determined by limiting dilution assays (TCID50 = tissue culture infective dose 50%) using fibroblasts as indicator cells and detection of viral immediate early antigens as a readout. (D) Supernatants of mutant and wild type virus were analyzed regarding loss of infectivity over time by repeated quantification of the infectivity at different time points after the harvest. Apparently, the viruses did not differ regarding the kinetics of decay of their biological activity. (TIF) [file pone.0131614.s001.tif]

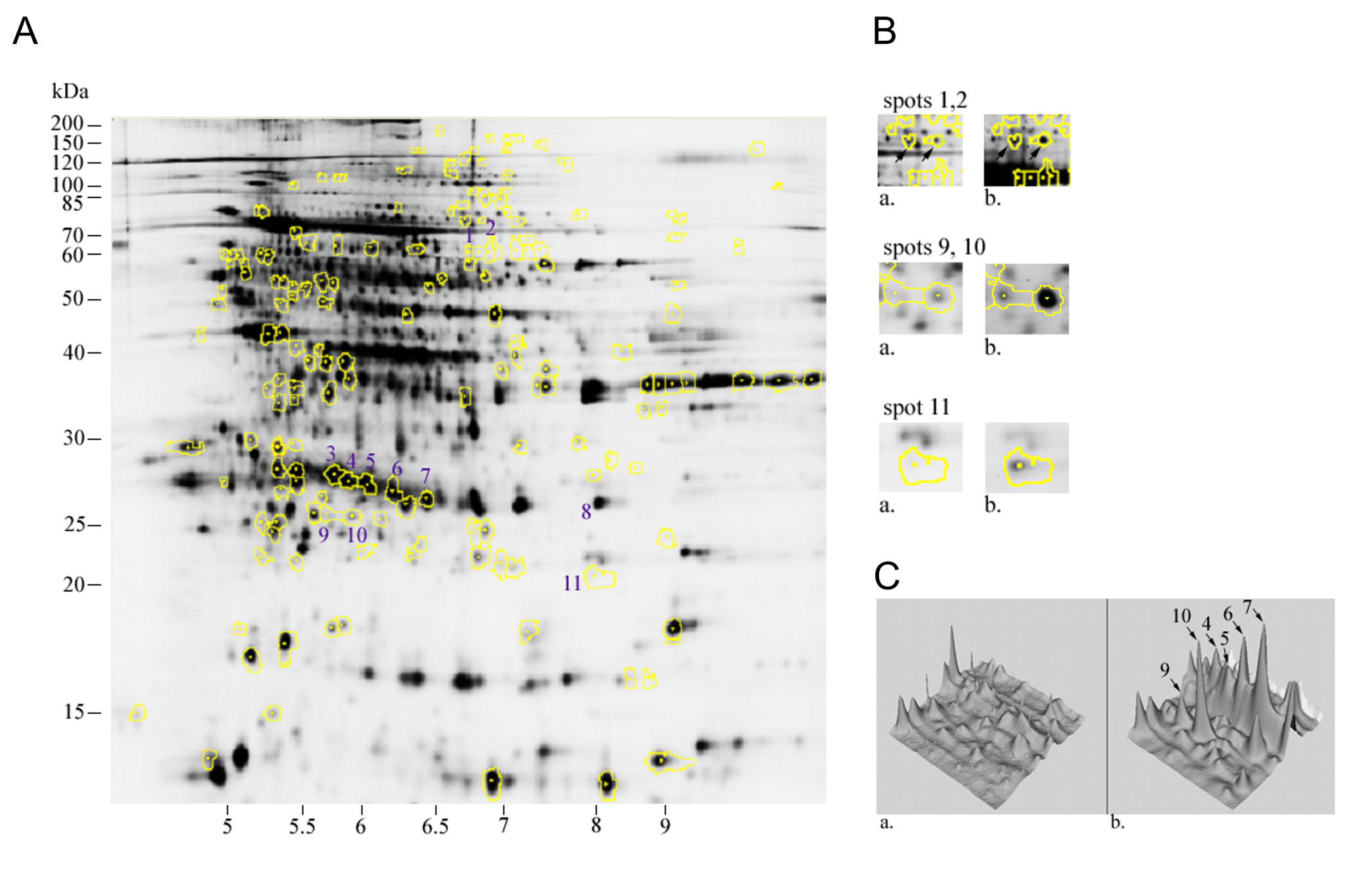

Supplement: S2 Fig — Protein lysates of gradient purified virions from TB40/E and the TB40/E-derived UL83-deletion mutant were labelled with Cy3 and Cy5, respectively. For an internal standard, lysates of both viruses were pooled and labelled with Cy2. A mixture of all three samples was run in 2D gel electrophoresis. (A) Indication of differentially represented spots as detected by Decyder analysis. (B) Interstrain comparison of spots with significant differences in the Decyder analysis. a = UL83 deletion virus, b = wild type virus. (C) Three-dimensional presentation of spots 3–7 (UL99) and spots 9–10 (Grb2) demonstrates the strong differences in expression of these proteins. a = UL83 deletion virus, b = wild type virus. (TIF) [file pone.0131614.s002.tif]

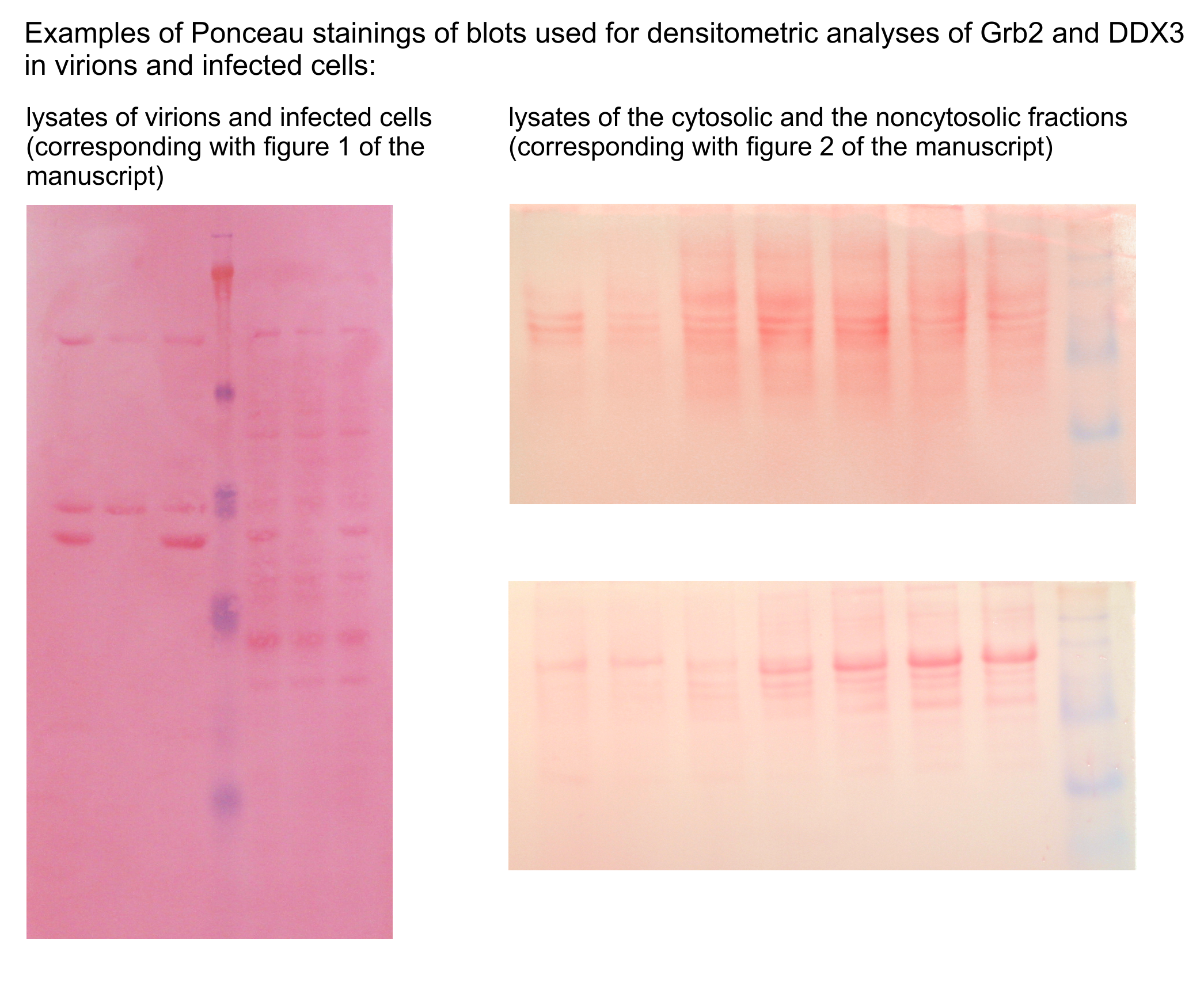

Supplement: S3 Fig — Blots were stained with Ponceau S and the protein content of each lane was evaluated by densitometric analysis to serve as a reference value for normalization of western blot signals. Examples of blots used for the generation of figs 1 and 2 of the manuscript are shown here. (TIF) [file pone.0131614.s003.tif]

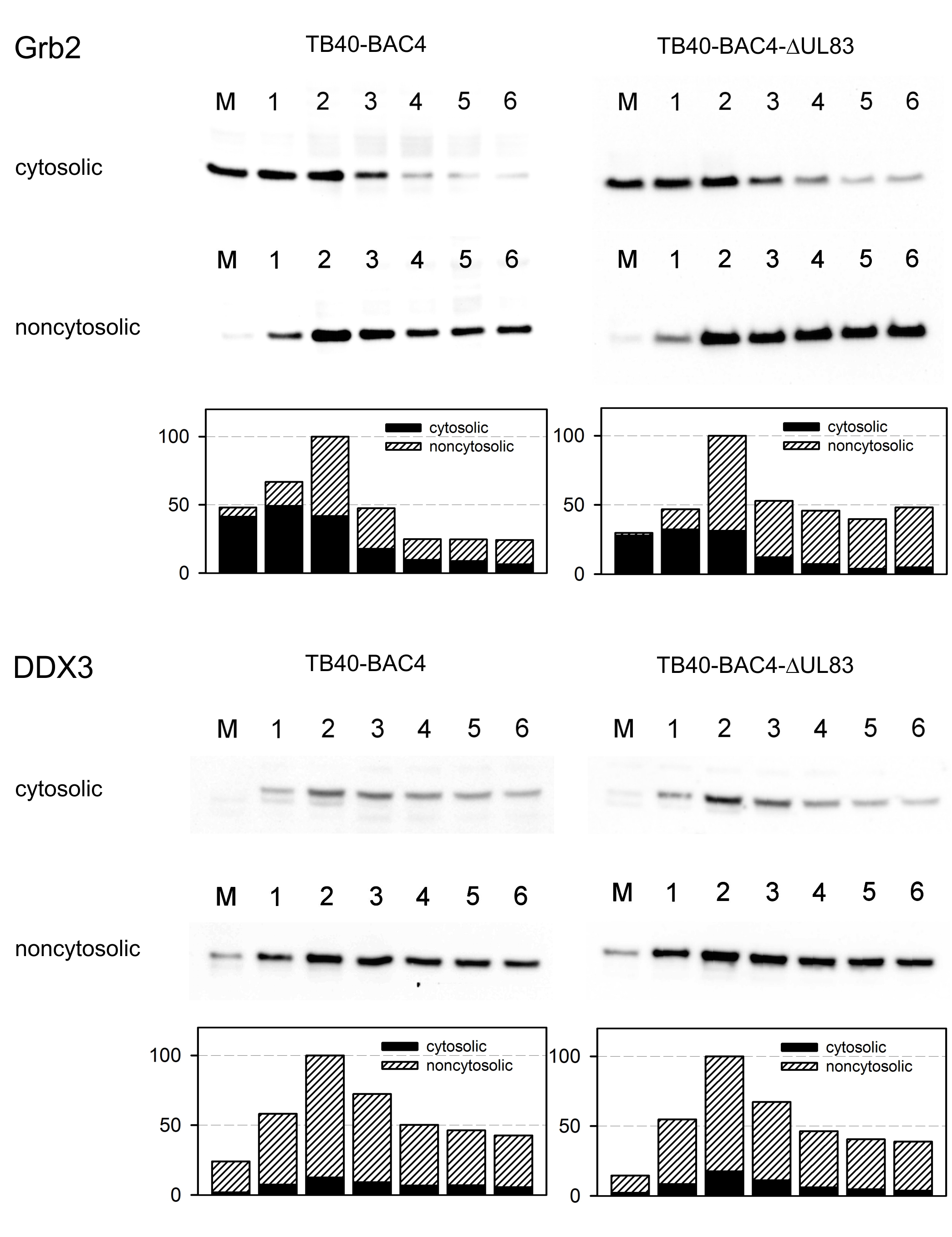

Supplement: S4 Fig — Cells were infected with wild type virus (TB40-BAC4) or a UL83-deletion mutant, and lysed with a mild detergent at 1–6 days after infection (d p.i. indicated by lane numbers), yielding a soluble cytosolic fraction and a residual nonsoluble fraction. Both fractions were further lysed with Lämmli buffer and analyzed by western blot for levels of DDX3 and Grb2. Western blot signals were analyzed by densitometry and normalized to the global amount of protein in the respective lane as determined by densitometric analysis of the Ponceau S staining. Mock-infected cells (M) were included as controls. For quantification, arbitrary units are given, relative to peak levels. (TIF) [file pone.0131614.s004.tif]

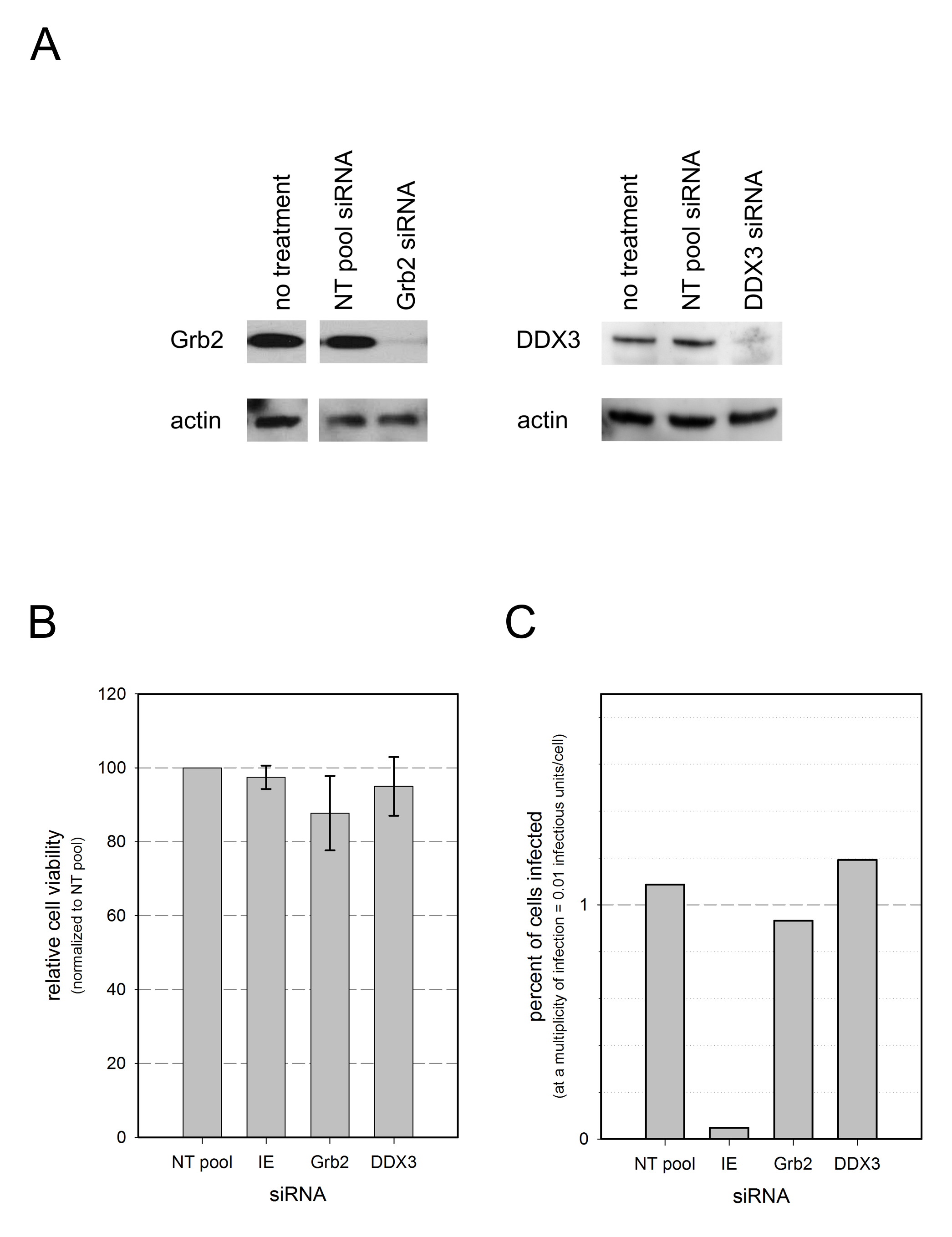

Supplement: S5 Fig — (A) HFFs were transfected with 50 nM siRNA targeting viral immediate early genes Ul122/123, cellular genes Grb2 and DDX3 or a pool of non-targeting (NT) siRNAs using Lipofectamine RNAiMAX transfection reagent (Life Technologies) and infected with HCMV strain TB40/E 48 hours after transfection at an approximate MOI of 1. At 5 d postinfection (p.i.) virus progeny was harvested. To control for the efficiency of the siRNA-mediated knockdown, cells were lysed and analyzed by western blotting under denaturing reducing conditions. (B) At 2 days after transfection of the respective siRNAs, cell viability was analyzed by measuring the metabolic activity of the cell cultures with the CellTiter-Blue Cell Viability Assay (Promega), using the indicator dye resazurin which is reduced to the highly fluorescent compound resorufin by metabolically active viable cells. (C) To test the effect of the respective siRNAs on infection efficiency, cells were infected at 2 days after transfection at an infection multiplicity of 0.01 infectious units (IU) per cell. At 1 day p.i., cells were stained for viral immediate early antigens by indirect immunofluorescence. (TIF) [file pone.0131614.s005.tif]
