# Supplementary material for: The Cellular Proteins Grb2 and DDX3 Are Increased upon Human Cytomegalovirus Infection and Act in a Proviral Fashion
Source: PLoS One. 2015 Jun 29;10(6):e0131614. doi: 10.1371/journal.pone.0131614 (PMC4509573; doi:10.1371/journal.pone.0131614)
Supplement: S1 Table — The protein patterns of delUL83-mutant and wild type virions were compared in a DIGE analysis. Virions were harvested from infected HFF and purified in glycerol-tartrate gradients. After lysis with 1% SDS, proteins were purified using the 2D clean up kit (GE Healthcare Bio-Sciences; "GE Healthcare"). Protein samples of mutant and wild type virions were resuspended in DIGE buffer (7 M urea, 2 M thiourea, 4% CHAPS, 30 mM Tris) at 2 mg/ml and labelled with Cy3 and Cy5 and vice versa (GE Healthcare). Internal pooled standard was prepared from equal amounts of each sample and labelled with Cy2. For each gel, 50 μg aliquots of Cy2-, Cy3- and Cy5-labelled samples were pooled and the final volume was adjusted to 460 μl with rehydration buffer (7 M urea, 2 M thiourea, 2% CHAPS, 1% DTT, 2% Pharmalyte, pH 3–10) and loaded on pH 3–10 gel strips (Immobiline DryStrips, GE Healthcare). Following overnight rehydration, isoelectric focusing was done in an IPGphor focusing unit for a total of 65.5 kVh. Gel strips were subsequently equilibrated twice for 15 min in equilibration buffer containing 6 M Urea, 30% glycerol (Sigma), 2% w/v SDS (Serva) and a trace of bromophenol blue, supplemented with 5 mg/ml DTT (Biorad) or 45 mg/ml iodacetamide (Fluka), respectively. Proteins were then separated in 12.5% SDS-PAGE gels using the DALTsix electrophoresis system (GE Healthcare). Gels were scanned with a Typhoon 9410 Variable Imager (GE Healthcare) and image analysis was performed with Decyder Differential Analysis Software version v7.5 (GE Healthcare). Parameters for the spot detection algorithm were set to “slope” 3 and “estimated number of spots” = 2000. For statistical analysis, the Student’s t-test p value was set to 0.05 with an average ratio of spots greater than 1.5 times intensity. Virion samples of both viruses from three independent gradient purifications were compared in three DIGE gels. Deletion of UL83 had a dramatic impact on the protein composition of the virion. Reproducible d [file pone.0131614.s006.pdf]

S3 Table: Characteristics of proteins found differentially represented between virions of TB40/E wild type and the respective pp65 deletion mutant

DeCyder analysis		Mr / pI	Mr / pI	mass spectrometric analysis			accession	protein
wt/mut	t-test	predicted	in gel	seq cov	peptides	mascot		
6.59	0.00025	25.1 / 6.1	25.1 / 5.9	15.20	4	125.0	gi|47496673	Grb2
5.05	0.005	73.0 / 7.2	73.0 / 6.7	3.20	2	67.8	gi|2580556	DDX3Y
3.70	0.042	25.1 / 6.1	25.1 / 5.6	32.71	9	471.2	gi|47496673	Grb2
3.50	0.004	21.1 / 9.1	20.0 / 8.0	24.46	6	273.7	gi|29423456	UL26 (HCMV)
3.07	0.022	73.2 / 6.8	73.0 / 6.8	25.52	18	768.6	gi|87196351	DDX3X
2.96	0.021	23.9 / 9.5	28.0 / 8.6	18.77	6	338.8	gi|62896867	CHMP2B
2.96	0.021	27.0 / 7.9	28.0 / 8.6	15.57	6	213.6	gi|6166390	CYB5R2
2.24	0.032	20.9 / 9.0	27.3 / 6.0	22.10	5	229.9	gi|39842106	UL99 (HCMV)
2.20	0.0078	21.8 / 9.9	27.5 / 8.0	8.16	2	73.7	gi|39644911	CHMP1.5
2.20	0.0078	26.2 / 9.0	27.5 / 8.0	10.52	3	129.4	gi|41350397	CACYBP
2.19	0.044	20.9 / 9.0	27.5 / 6.2	10.00	2	43.5	gi|39842106	UL99 (HCMV)
2.06	0.022	20.9 / 9.0	27.0 / 6.4	10.00	2	46.6	gi|39842106	UL99 (HCMV)
1.95	0.07	20.9 / 9.0	28.5 / 5.5	14.21	3	110.0	gi|39842106	UL99 (HCMV)
1.92	0.044	20.9 / 9.0	27.5 / 5.8	22.10	5	178.3	gi|39842106	UL99 (HCMV)
1.89	0.030	37.5 / 5.9	38 .0/ 5.9	3.05	1	48.3	gi|54696354	PP1
1.88	0.041	45.7 / 4.9	52.0 / 4.9	12.46	4	127.6	gi|28373252	UL71 (HCMV)
1.73	0.019	21.7 / 6.3	23.0 / 6.4	24.48	5	226.2	gi|20531765	CLDD6. hypothetical protein
1.67	0.027	16.8 / 4.9	17.0 / 5.2	6.52	2	46.5	gi|12653915	eIF-5A
1.60	0.047	21.6 / 6.2	25.1 / 6.1	21.69	5	223.5	gi|55962165	RHOC
1.55	0.015	68.4 / 5.0	76.0 / 5.3	20.80	13	770.1	gi|81097637	cytoplasmic dynein intermediate
-1.78	0.00027	47.2 / 4.8	49.0 / 4.9	14.50	6	305.4	gi|12803811	ENO2. Enolase 2
-1.92	0.044	38.6 / 8.5	37.0 / 5.3	17.40	6	225.0	gi|18645167	Annexin A2
-2.33	0.0016	29.9 / 4.6	27.0 / 5.5	27.92	9	477.5	gi|49119653	YWHAZ
-2.65	0.0023	29.4 / 5.4	29.5 / 5.4	19.01	5	271.2	gi|40226101	Actin beta
-2.69	0.00005	29.8 / 5.5	28.0 / 5.5	19.11	12	493.3	gi|49456373	PHB. prohibitin
-2.75	0.0027	41.0 / 5.5	40.0 / 5.8	23.36	17	697.6	gi|14250401	Actin beta
-2.99	0.02	36.0 / 9.3	37.0 / 8.8	6.56	2	49.0	gi|31645	GAPDH
-3.35	0.00048	13.8 / 8.9	12.5 / 8.9	31.50	4	148.7	gi|825616	124 aa fragment of beta actin
-3.44	0.0015	41.0 / 5.5	40 .0/ 5.7	7.75	2	64.8	gi|14250401	Actin beta
-4.26	0.042	15.1 / 9.7	11.5 / 6.8	25.53	3	90.1	gi|1335076	Alpha-2-globin (cleavage product)
-4.57	0.0031	41.0 / 5.5	40.0 / 5.6	22.00	12	441.6	gi|14250401	Actin beta
-7.57	0.018	15.1 / 9.7	11.5 / 8.2	11.34	2	73.1	gi|1335076	Alpha-2-globin (cleavage product)

wt/mut = ratio of protein amount between wild type virions and mutant virions

Mr = relative molecular mass

pI = isoelectric point

Seq cov = sequence coverage [%]

Peptide = number of unique peptides

Mascot = mascot score

(HCMV) = viral protein
